# Histological and Functional Benefit Following Transplantation of Motor Neuron Progenitors to the Injured Rat Spinal Cord

**DOI:** 10.1371/journal.pone.0011852

**Published:** 2010-07-29

**Authors:** Sharyn L. Rossi, Gabriel Nistor, Tanya Wyatt, Hong Zhen Yin, Aleksandra J. Poole, John H. Weiss, Matthew J. Gardener, Sipke Dijkstra, David F. Fischer, Hans S. Keirstead

**Affiliations:** 1 Department of Anatomy & Neurobiology, School of Medicine, Reeve-Irvine Research Center, Sue and Bill Gross Stem Cell Research Center, University of California at Irvine, Irvine, California, United States of America; 2 Department of Neurology, University of California at Irvine, Irvine, California, United States of America; 3 California Stem Cell, Inc., Irvine, California, United States of America; 4 BioFocus, a Galapagos company, Chesterford Research Park, Saffron Walden, United Kingdom; 5 Galapagos, Leiden, The Netherlands; 6 BioFocus, a Galapagos company, Leiden, The Netherlands; Brigham and Women's Hospital, Harvard Medical School, United States of America

## Abstract

**Background:**

Motor neuron loss is characteristic of cervical spinal cord injury (SCI) and contributes to functional deficit.

**Methodology/Principal Findings:**

In order to investigate the amenability of the injured adult spinal cord to motor neuron differentiation, we transplanted spinal cord injured animals with a high purity population of human motor neuron progenitors (hMNP) derived from human embryonic stem cells (hESCs). *In vitro*, hMNPs displayed characteristic motor neuron-specific markers, a typical electrophysiological profile, functionally innervated human or rodent muscle, and secreted physiologically active growth factors that caused neurite branching and neuronal survival. hMNP transplantation into cervical SCI sites in adult rats resulted in suppression of intracellular signaling pathways associated with SCI pathogenesis, which correlated with greater endogenous neuronal survival and neurite branching. These neurotrophic effects were accompanied by significantly enhanced performance on all parameters of the balance beam task, as compared to controls. Interestingly, hMNP transplantation resulted in survival, differentiation, and site-specific integration of hMNPs distal to the SCI site within ventral horns, but hMNPs near the SCI site reverted to a neuronal progenitor state, suggesting an environmental deficiency for neuronal maturation associated with SCI.

**Conclusions/Significance:**

These findings underscore the barriers imposed on neuronal differentiation of transplanted cells by the gliogenic nature of the injured spinal cord, and the physiological relevance of transplant-derived neurotrophic support to functional recovery.

## Introduction

Cell based strategies for central nervous system (CNS) repair are inherently combinatorial in nature, in that they replace dead or dying cells, provide a substrate for endogenous neural growth and angiogenesis, and provide neurotrophic support for the endogenous environment. The contribution of a cell type's phenotypic or neurotrophic benefit to functional recovery has historically been difficult to distinguish, as the heterogeneous population of cells within most transplants yields multiple cellular phenotypes and neurotrophic secretions post-transplantation.

Neurotrophic secretions characterize all progenitor cells and are known to alter injury and disease pathogenesis. For example, human neural stem cells constitutively secrete nerve growth factor (NGF), brain-derived neurotrophic factor (BDNF), and glial-derived neurotrophic factor (GDNF) that promote growth of injured axons following spinal cord injury [1]. Human embryonic germ cell-derived embryoid bodies secrete BDNF and transforming growth factor α (TGF-α) that enhance neuronal survival and reafferentation of host motor neurons when transplanted into rats with diffuse motor neuron paralysis [2]. In addition, oligodendrocyte progenitors differentiated from hESCs express hepatocyte growth factor (HGF), Activin A, transforming growth factor β-2 (TGF-β2) and BDNF [3], which may contribute to recovery of function following SCI [4], [5]. Thus, transplanted stem cell derivates can influence the injured environment by providing survival factors, guidance molecules, or cues for proliferation and differentiation of endogenous stem and progenitor cells.

Likewise, the differentiation of transplanted cells is dictated by the environmental signals and cellular deficiencies operating at the site of implantation. Transplantation of multipotent spinal cord progenitor cells into the uninjured spinal cord results in glial differentiation [6], [7], while transplantation into neurogenic regions of the brain results in neuronal differentiation [6]. Within the injured spinal cord, the innate gliogenic repair mechanisms enhance glial differentiation of transplanted multipotent cells. This is evidenced by multiple studies demonstrating glial differentiation of neural stem cells [8], adult spinal cord progenitor cells [7], [9], [10] and human embryonic stem cell-derived oligodendrocyte progenitor cells [5] following transplantation into injured spinal cords. Indeed, neuron-restricted precursor cells can differentiate into mature neuronal subtypes when transplanted into adult non-injured spinal cord, but retain an immature state when transplanted into injured spinal cord [11].

In order to investigate the reciprocal interaction between the injured spinal cord and a motor neuron progenitor transplant population, we treated spinal cord injured animals with a high purity population of hMNPs. Our data underscore the gliogenic nature of the injured adult spinal cord, and the physiological relevance of transplant-derived neurotrophic factor release.

## Results

### Summary of Experimental Design

hESCs were differentiated into high purity populations of hMNPs. The phenotype of the resulting cell population was confirmed by analyses of morphology, immunocytochemical marker expression, kainate-stimulated uptake of Co^2+^ ions by Ca^2+^ permeable AMPA channels, electrophysiology, glutamate-mediated excitotoxicity, and hMNP-muscle co-cultures. To investigate the neurotrophic potential of hMNP secretions *in vitro*, we performed growth factor expression analyses, followed by functional assays of hMNP secretions on cortical neurons to assess neurite branching, axonal regeneration and survival of cortical neurons in a neurotoxic environment. The neurotrophic activity of hMNP secretions was confirmed by exposing cortical neurons to hMNP-CM pre-incubated with function blocking antibodies to hMNP-secreted growth factors. The survival, differentiation, and site-specific integration of hMNPs transplanted in the ventral horns cranial and caudal to bilateral cervical contusion injuries was investigated at distances from the injury sites, to investigate the reciprocal interaction between the injured spinal cord and a neuronal transplant population. A rodent model of motor neuron loss was also transplanted with hMNPs, to ensure that the differentiation profile in spinal cord injured animals was a result of the injured spinal cord rather than an inherent property of the transplant population. To investigate the neurotrophic potential of hMNP secretions *in vivo*, we assayed transplanted animals for sprouting of endogenous fiber tracts, sparing of endogenous neurons, gross tissue sparing, suppression of intracellular signaling pathways associated with SCI pathogenesis, and functional ability.

### hMNP Differentiation

**Figure 1:**
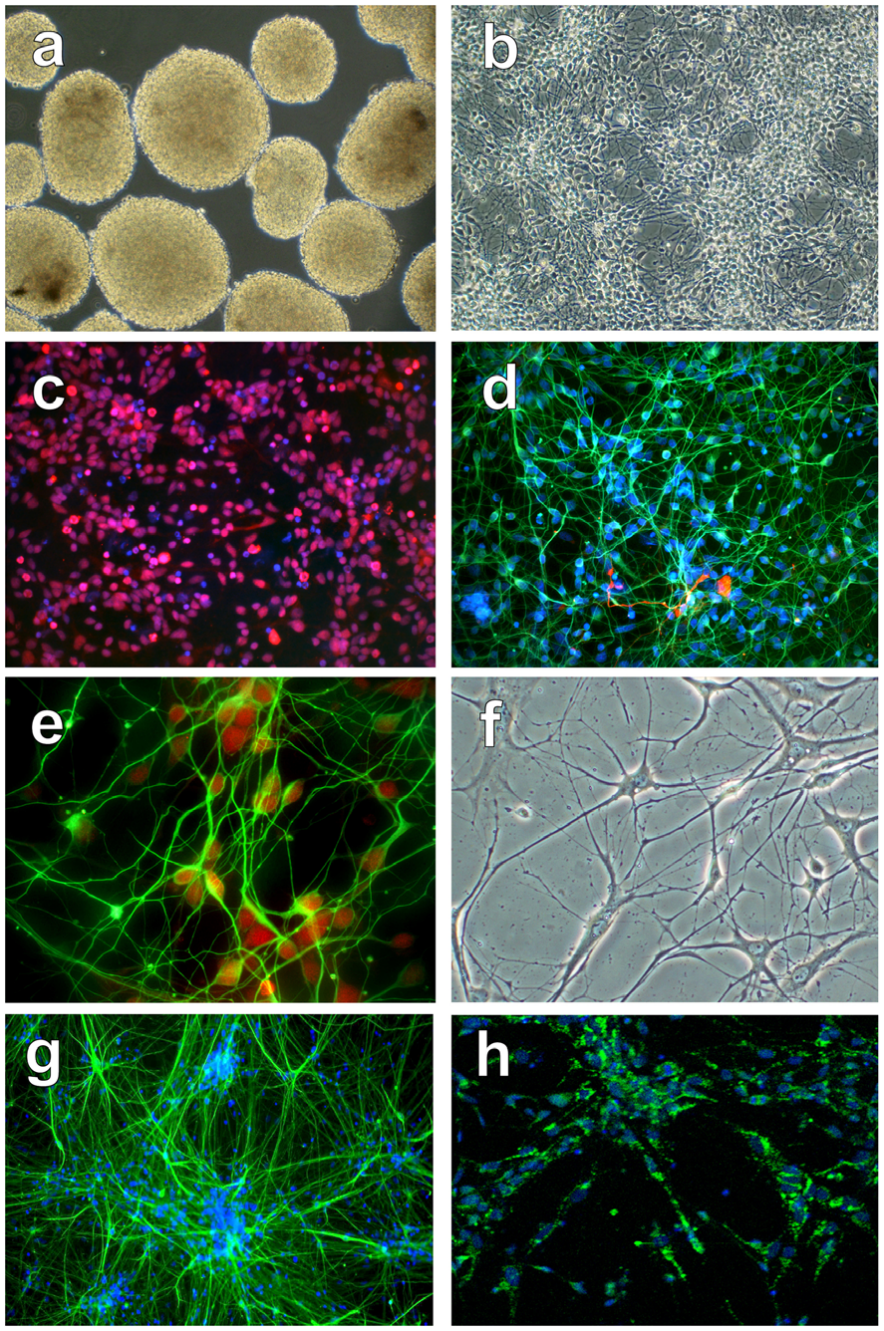
Morphological and immunocytochemical characterization of hMNPs at different stages of differentiation. (a) At day 7 of the 28 day protocol, cultures contained yellow solid core neurospheres in the absence of single floating cells. (b) At day 25, morphologically homogeneous cells exhibited processes, and (c) expressed the MN lineage marker Olig 1/2 (red), and (d) Tuj1 (green); GFAP+ astrocytes (red) were rare within these Tuj1+ cultures. Nuclear counterstain for (c) and (d) is blue. (e) At day 28, the majority of Tuj1+ cells (green) double stained for the MN lineage marker HB9 (red). (f) After 3 weeks of subsequent growth, cells displayed a mature, branched morphology consistent with mature MNs, and expressed the mature MN markers (g) SMI-32 and (h) ChAT. Bar = 1000 µm for (a), 200 µm for (b) and (g), 100 µm for (c) and (d), 50 µm for (e), 33 µm for (f), and 150 µm for (h).

At day 7 of the 28 day differentiation protocol, 47.8+/−18.2% of cells expressed the undifferentiated hESC marker OCT4, 42.5+/−13% of cells expressed the early neural progenitor marker Pax6, 38.3+/−12.1% of cells expressed the early neural progenitor marker nestin, 4.8+/−2% of cells expressed the mesodermal marker SMA, and 0.5+/−1.3% of cells expressed the mesodermal marker aFP. Cultures consisted of solid core 100–600 µm spheres (Fig. 1a), and single cells were eliminated by daily feeding. After plating at day 21, hMNPs migrated radially from neurospheres.

At day 25, cells exhibited processes (Fig. 1b) and 99. 2+/−0.6% of cells expressed the MNP marker Olig1/2 (Fig. 1c), 99.2+/−0.44% of cells expressed the MNP marker Isl-1, 98.8+/−1.3% of cells expressed the neuronal marker Tuj1 (Fig. 1d, green). Less than 1% of cells expressed the astrocyte marker GFAP (Fig. 1d, red), 5.5%+/−3.6% of cells expressed the MN marker HB9, and no cells expressed the mature MN marker ChAT. Less than 0.1% remained unlabeled.

At day 28, the day of transplantation, 96.7+/−2.62% of cells expressed the MN marker HB9 (Fig. 1e). Similar to the day 21 immunocytochemical profile, less than 1% of cells expressed the astrocyte marker GFAP and no cells expressed the mature MN marker ChAT. No Oct4 positive stem cells could be identified on or after day 28.

To assess maturation in vitro, hMNPs were plated on astrocytes and allowed to mature for 3 weeks. Matured hMNPs displayed a mature, branched morphology (Fig. 1f), 97.7+/−1.53% of cells expressed the mature neurofilament marker SMI-32 (Fig. 1g), and 96.6+/−4.4% of cells expressed the MN marker ChAT (Fig. 1h), while Olig 1/2 and HB9 expression decreased significantly (p<0.001; 2+/−1.3% and 6+/−4.4%, respectively). SMI-32 positive cells exhibited distinct morphological features of mature MNs, including a large cell body size (>25 µm), a well-developed dendritic tree, and a distinct axon. MN maturation was further demonstrated by kainate-stimulated uptake of Co^2+^ ions by Ca^2+^ permeable AMPA channels. Less than 1% of anti-human nuclear positive cells displayed GFAP+ immunoreactivity, and no SMI-32, or ChAT immunoreactive cells were GFAP+. Less than 0.1% remained unlabeled.

### MN Functionality

**Figure 2:**
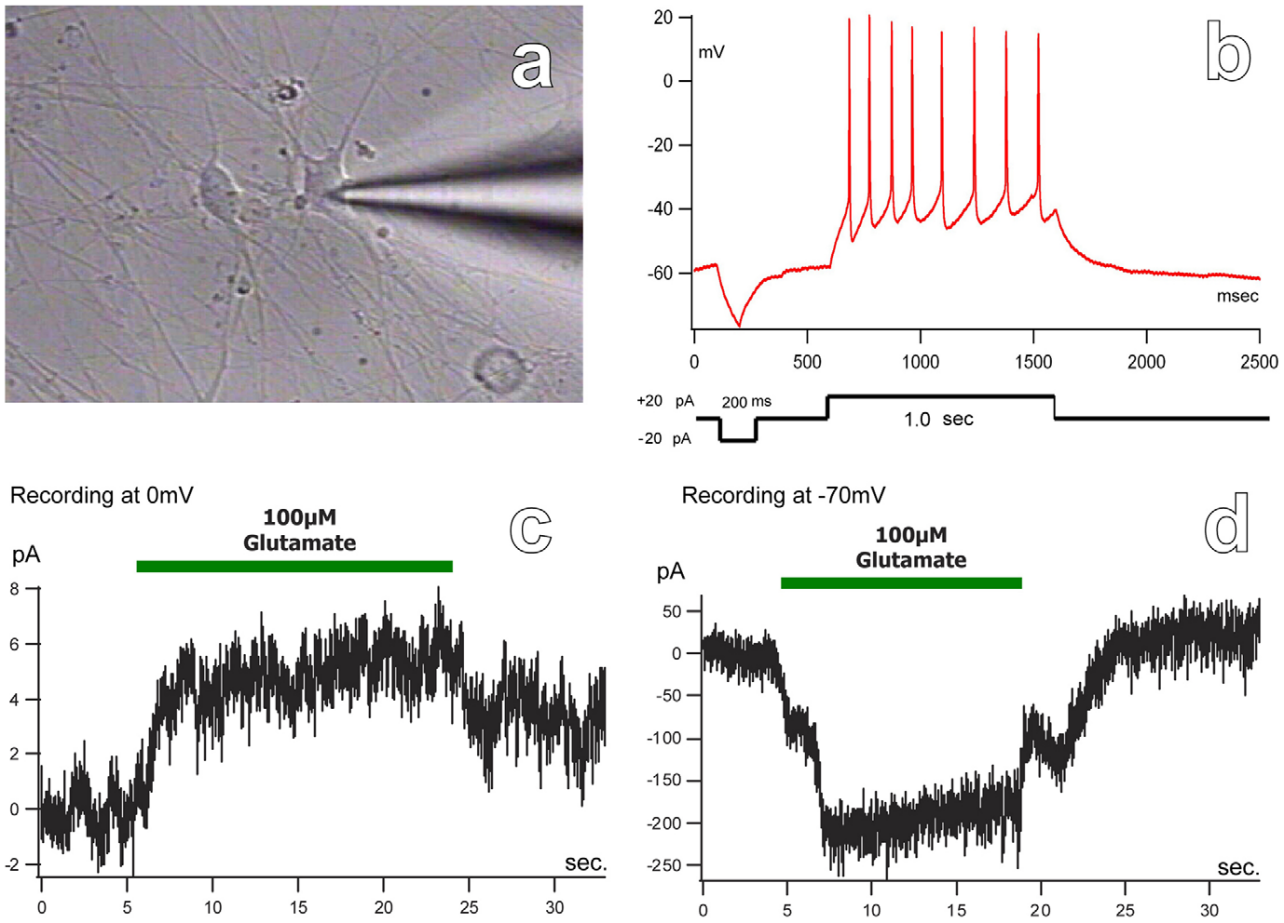
Electrophysiological profile of hESC-derived MNs. (a) Electrophysiological activity was assessed in MNs current clamped in the whole cell configuration. (b) Injection of 20 pA of current elicited action potential trains, typical of mature MNs. The presence of glutamate receptors was evidenced using symmetrical solutions. (c) At 0 mV, the addition of glutamate mediated a small outward current, likely due to the presence of KOH in the internal solution. (d) At −70 mV, the addition of glutamate mediated a large inward current, as K^+^ and Ca^2+^ ions flowed through open glutamate receptors. Bar = 33 µm.

The functionality of hMNPs was assessed by electrophysiology, glutamate-mediated excitotoxicity, and hMNP-muscle co-culture experiments. hMNPs matured for 8 weeks consistently displayed resting potentials of −40 to −60 mV. Injection of 20pA to MNs current clamped in the whole cell configuration (Fig. 2a) elicited trains of action potentials, typical of mature MNs (Fig. 2b). The presence of glutamate receptors was evidenced using symmetrical solutions such that at 0 mV, the addition of 100 µM glutamate mediated a small outward current, likely due to the presence of KOH in the internal solution (Fig. 2c), and at −70 mV the addition of glutamate mediated a large inward current, as K^+^ and Ca^2+^ ions flowed through open glutamate receptors (Fig. 2d).

Exposure of hMNPs to glutamic acid at increasing concentrations resulted in increasing amounts of hMNP death. The percentage of propidium iodide (PI) positive cells was 11.36+/−1.25% for 0 µM glutamic acid, 9.68+/−2.2% for 500 µM glutamic acid, and 17.47+/−1.3% for 2000 µM glutamic acid. The percentage of Annexin V positive cells was not significantly different (p>0.01) than the percentage of PI positive cells.

Separation of MN and muscle cell bodies in microfluidic culture platforms connected by 5–20 µm micro-channels [12] was required for stable functional innervation of myotubes. hMNPs progressively matured, as evidenced by an enlarged cell body size, an increase in dendritic arborization, and extension of neurites through the micro-channels into the other chamber. Approximately 40% of the myoblasts fused to form myotubes, which displayed spontaneous contractile activity upon innervation with MN neurites. After innervation, α-bungarotoxin, synaptophysin and ChAT staining was significantly (p<0.001) increased over myotube cultures that lacked innervation. No spontaneous contractile activity, α-bungarotoxin, synaptophysin and ChAT staining could be detected in this system when no hMNPs were seeded.

### hMNPs Secrete Physiologically Active Growth Factors

**Figure 3:**
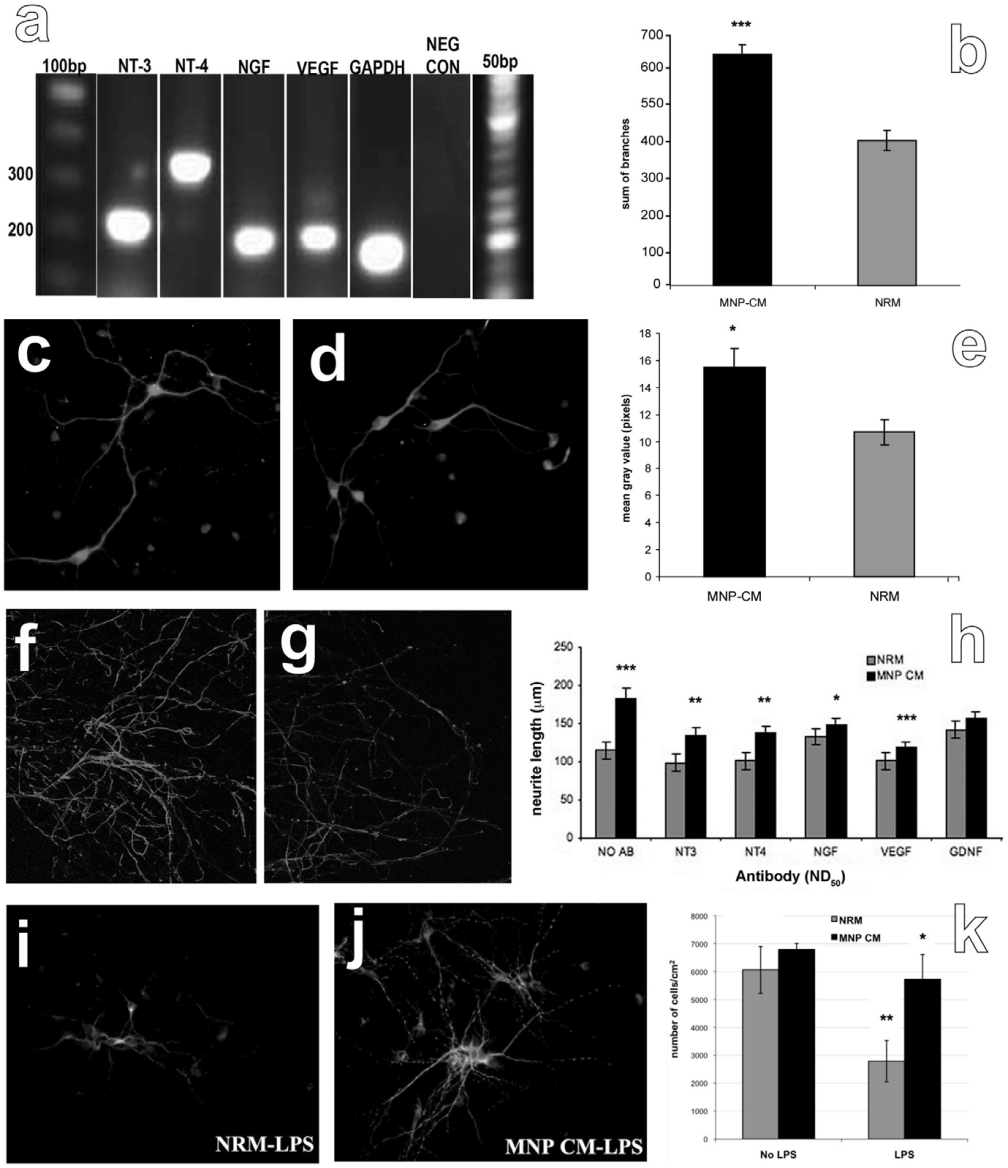
hMNPs secreted physiologically active growth factors. (a) Qualitative PCR analyses indicated that hMNPs express NT-3, NT-4, NGF, and VEGF. Neurite length was 58% longer (b) in cortical neuron cultures exposed to hMNP-CM for 7 days (c) as compared to cortical neuron cultures exposed to MN differentiation media (d). The neurofilament optical density was 45% greater (e) in the axonal chamber of microfluidic culture platforms in axotomized cortical neuron cultures exposed to hMNP-CM for 7 days (f) as compared to axotomized cortical neuron cultures exposed to MN differentiation media (g). (h) Neurite length was significantly attenuated in cortical neuron cultures exposed to hMNP-CM that contained function-blocking antibodies to MN growth factors. Immunofluorescent staining for MAP-2 in cultures exposed to control media (i) or hMNP-CM (j), in the presence of LPS. (k) Quantification of the number of MAP-2 positive neurons in the presence of control media or hMNP-CM, with and without LPS exposure. The number of neurons in cultures lacking LPS was not significantly different (p<0.05) in cultures exposed to control media or MNP CM, however, the number of neurons in cultures with LPS was significantly higher (p<0.05) in cultures exposed to hMNP-CM as compared to those exposed to control media. Bar = 50 µm.

To investigate the neurotrophic potential of hMNP secretions, we performed growth factor expression analyses, followed by functional assays of hMNP secretions on cortical neurons *in vitro*. PCR (Fig. 3a) and Western blot analyses (Fig. S1b) indicated that hMNPs both express and secrete neurotrophin-3 (NT-3), neurotrophin-4 (NT-4), nerve growth factor (NGF), and vascular endothelial-derived growth factor (VEGF). Further analysis using real-time PCR revealed a 10-fold increase in NT-3, a 23-fold increase in NT-4, an 18-fold increase in VEGF, a 3.5-fold increase in CNTF (ciliary neurotrophic factor), a 6-fold increase in IL10, a 121-fold increase in NRG4 (neuregulin 4), and a 275-fold increase in NPY (neuropeptide Y) when compared to hFib controls. Interestingly, there was a 5-fold decrease in NGF expression in hMNP when compared to hFibs (Fig. S1a). PCR analyses indicated that TGFα, BDNF and GDNF were not produced by the hMNPs.

Neurite length was 58% longer (Fig. 3b) in cortical neuron cultures exposed to hMNP-CM for 7 days (Fig. 3c) as compared to cortical neuron cultures exposed to MN differentiation media (Fig. 3d; 182+/−15 µm and 115+/−11 µm, respectively; p<0.001). The neurofilament optical density was 45% greater (Fig. 3e) in the axonal chamber of microfluidic culture platforms in axotomized cortical neuron cultures exposed to hMNP-CM for 7 days (Fig. 3f) as compared to axotomized cortical neuron cultures exposed to MN differentiation media (Fig. 3g; 15.5+/−2.2 µm and 10.7+/−1.8 µm, respectively; p<0.001). MAP-2 immunostaining of the two chamber microfluidic culture platforms confirmed that no cells migrated to the axonal chamber.

The neurotrophic activity of hMNP secretions was confirmed by exposing cortical neurons to hMNP-CM pre-incubated with function blocking antibodies to hMNP-secreted growth factors. Neurite length was significantly attenuated in cortical neuron cultures exposed to hMNP-CM that contained function-blocking antibodies to hMNP-secreted growth factors (Fig. 3h); average neurite length decreased from 185+/−10 µm to 135+/−9 µm in the presence of anti-NT3 (p<0.01), 138+/−8 µm in the presence of anti-NT4 (p<0.01), 149+/−8 µm in the presence of anti-NGF (p<0.01), and 119+/−6 µm in the presence of anti-VEGF (p<0.01). There was no significant difference (p>0.01) in neurite length of cortical neurons cultured in the presence of hMNP-CM containing function-blocking antibodies to GDNF, which is not detected in hMNPs (156+/−10 µm). In addition, there was no significant difference (p>0.01) in average neurite length of cortical neurons cultured in the presence of MN differentiation media (115+/−11 µm) and cortical neurons cultured in the presence of MN differentiation media containing function-blocking antibodies to NT3 (99+/−7 µm), NT4 (101+/−9 µm), NGF (133+/−17 µm), VEGF (102+/−6 µm), and GDNF (142+/−9 µm).

To investigate the effects of hMNP-CM on neuronal survival in a neurotoxic environment, cortical neurons were exposed to microglial conditioned media (MG CM) or LPS-activated microglial conditioned media (LPS MG-CM), in the presence of hMNP-CM or motor neuron differentiation media. Immunofluorescent staining for MAP-2 in cultures exposed to control media (Fig. 3i) or hMNP-CM (Fig. 3j) in the presence of MG-CM, indicated that the number of MAP-2 positive neurons in cultures without LPS activation was not significantly different (p<0.05). In cultures exposed to LPS MG-CM, however, the number of neurons was significantly higher (p<0.05) in the presence of hMNP-CM as compared to control media (Fig. 3k). These findings demonstrate that hMNPs secrete physiologically active growth factors which promote neuronal survival in an inflammatory environment and enhance axonal sprouting and regeneration *in vitro*.

### Localization and Differentiation of hMNPs in a SCI Model

**Figure 4:**
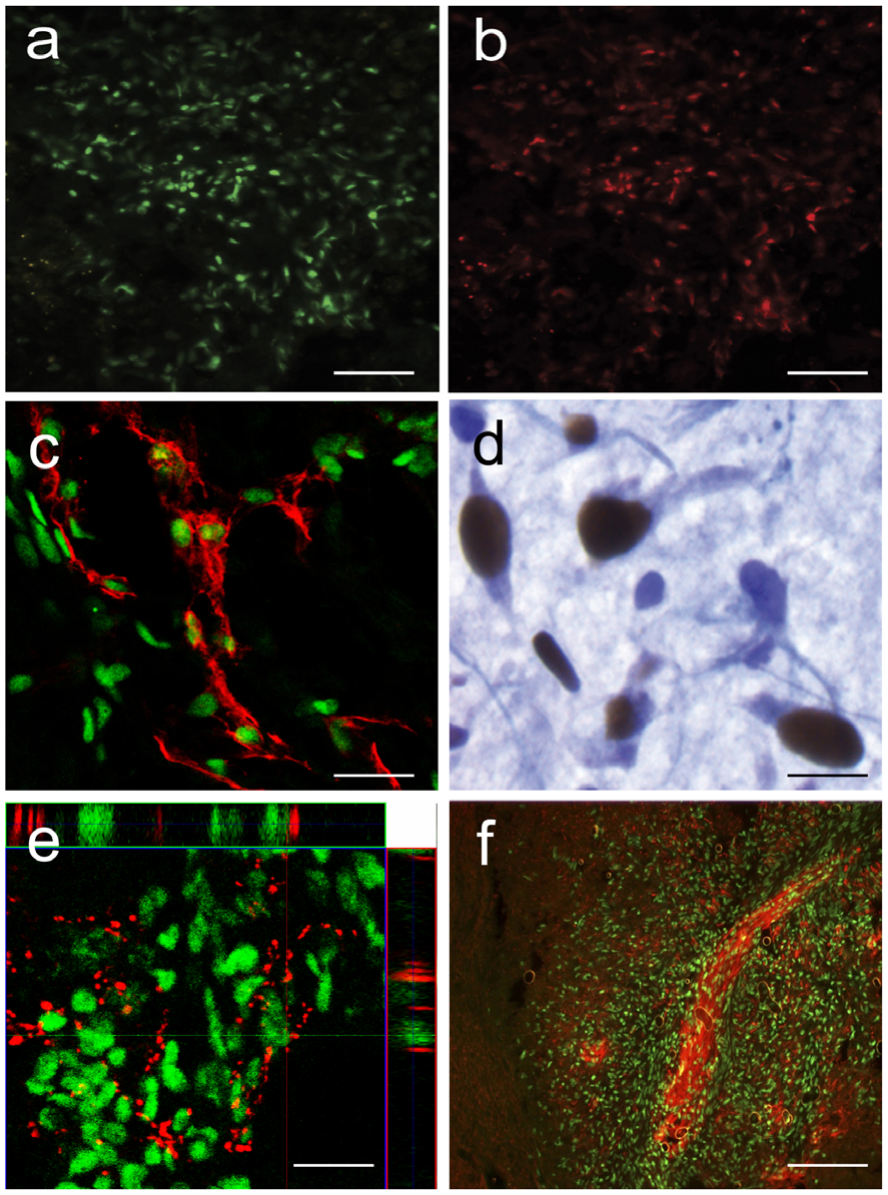
Transplanted hMNPs differentiated following transplantation. Human nuclear antigen-positive cells (a) double stained with Isl-1 (b; red), p75 (c; p75 in red, human nuclei in green), or ChAT (d; ChAT in blue, human nuclei in brown), consistent with a MN lineage of mixed maturation state. (e) Some human cells in the ventral horns were surrounded by synaptophysin positive processes, suggesting integration with host tissue (synaptophysin in red, human nuclei in green). (f) Many human cells extended Tuj1 positive processes, and in some animals, ectopic motor tracts were present in the dorsal and ventral white matter (Tuj1 in red, human nuclei in green). Bar = 100 µm for (a) and (b), 50 µm for (c) and (e), 10 µm for (d), 200 µm for (f).

To investigate the reciprocal interaction between the injured spinal cord and a neuronal transplant population, the survival, differentiation, and site-specific integration of hMNPs was investigated at distances from SCI sites, following transplantation of hMNPs cranial and caudal to bilateral contusion injuries. Human nuclear antigen-positive cells were detected in all transplanted animals, and did not migrate from the transplant sites cranial and caudal to the injury epicenter. Human cells (Fig. 4a) within the ventral horns double stained with Isl-1 (Fig. 4b), p75 (Fig. 4c; p75 in red, human nuclei in green), neurofilament or ChAT (Fig. 4d; ChAT in blue, human nuclei in brown), consistent with a MN lineage of mixed maturation state. Some human cells in the ventral horns were surrounded by synaptophysin positive processes, suggesting integration with host tissue (Fig. 4e; synaptophysin in red, human nuclei in green). Many human nuclear antigen-positive cells extended Tuj1 positive processes, and in some animals, ectopic motor tracts were present in the dorsal and ventral white matter (Fig. 4f; Tuj1 in red, human nuclei in green). Tuj1 positive structures had an irregular trajectory and included varicosities along their length, characteristic of new axon growth [13]. No-primary and no-secondary antibody controls yielded no Tuj1 tissue staining. None of the CTB+ MNs retrogradely labeled following CTB injection into peripheral muscle contained a human nucleus, demonstrating that transplanted cells did not extend axons into the periphery or form neuromuscular junctions with host tissue, as expected.

Although most transplanted cells were located in the parenchyma cranial and caudal to the injury epicenter where they were placed, transplanted cells were found within the injury epicenter in 5 out of 15 animals. These human cells outside of the ventral horns were predominantly nestin positive or double cortin positive, suggesting back-differentiation to an early neuronal phenotype, and some were GFAP positive suggesting amplification of the minor astrocyte population within the transplant. No human nuclear antigen-positive cells expressed markers for oligodendrocytes.

### Localization and Differentiation of hMNPs in a Motor Neuron Loss Model

The *Smn^−/−^;SMNΔ7^+/+^;SMN2^+/+^* model of motor neuron loss was also transplanted with hMNPs, to ensure that the differentiation profile in spinal cord injured animals was a result of the injured spinal cord rather than an inherent property of the transplant population. As these mutants have a shortened lifespan, animals were sacrificed within 13 days of transplantation; the lifespan of this animal model limits the time available for differentiation of transplanted cells. Human cells were detected in all *Smn^−/−^;SMNΔ7^+/+^;SMN2^+/+^* mice in the ventral horns of the spinal cord. All human nuclear antigen-positive cells double stained with Isl-1, confirming the MN differentiation potential of the transplant population in a model of MN loss that lacks a spinal cord injury. Isl-1 staining was absent in non-transplanted animals, consistent with their MN pathology. Human nuclear antigen-positive cells did not double label with markers for the mature motor neuron markers ChAT or SMI-32, indicating that 13 days of survival in vivo was insufficient for differentiation of transplanted hMNPs. Importantly, very few of the human-positive cells were nestin, double-cortin, or GFAP positive, indicating that the abundance of these cells in SCI sites was a result of the SCI environment rather than the default differentiation profile of the transplant population.

### Transplantation of hMNPs Caused Histological Benefit

To investigate the neurotrophic potential of hMNP secretions *in vivo*, we assayed transplanted animals for sprouting of endogenous fiber tracts, sparing of endogenous neurons, gross tissue sparing, and suppression of intracellular signaling pathways associated with SCI pathogenesis.

**Figure 5:**
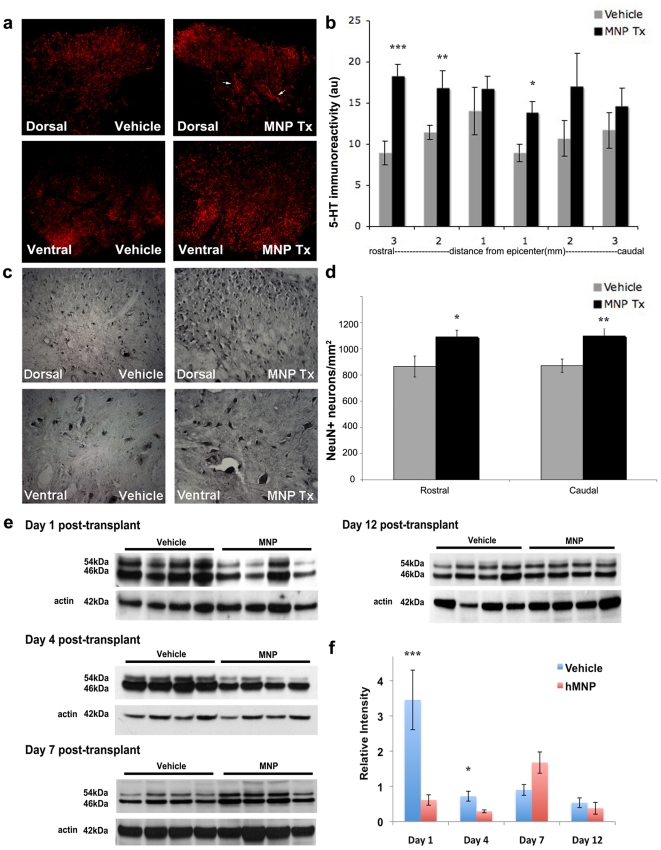
Transplanted hMNPs promote histological recovery and alter intracellular signaling pathways. (a) hMNP transplantation enhanced sprouting of endogenous serotonergic (5-HT) projections. hMNP-transplanted animals consistently contained aberrant projections throughout the dorsal gray matter (top panels, arrows) and dense innervation of the ventral horns (bottom panels) at 2 mm cranial to the injury site (b) At 2 mm and 3 mm cranial to the injury epicenter, and 1 mm caudal to the injury epicenter, 5-HT immunoreactivity was significantly greater than that observed in control animals. At 1 mm cranial to the injury epicenter, and 2 mm and 3 mm caudal to the injury epicenter, 5-HT immunoreactivity was not significantly different than in control animals. (c) NeuN immunostaining demonstrated that hMNP transplantation enhanced survival of endogenous (human nuclear antigen-negative) neurons 2 mm cranial to the injury site. (d) Quantification of enhanced neuronal survival in hMNP-transplanted animals cranial and caudal to the injury site. (e) hMNP transplantation attenuated phosphorylation of stress-associated protein kinase (SAPK). (f) Densitometric quantification of SAPK normalized to actin controls showed that 1 and 4 days following transplantation, phosphorylation of SAPK decreased in hMNP-transplanted animals relative to controls; no significant differences were observed between groups at 7 and 10 days. Bar = 200 µm for (a), 100 µm for (c).

hMNP transplantation enhanced sprouting of endogenous serotonergic (5-HT) projections (Fig. 5a, b). In vehicle-injected animals, the patterning of serotonergic projections was consistent with that documented in the literature; there was high innervation of laminae I and II, some diffuse labeling in lamina V, and highly distributed innervation throughout the ventral horn (Fig. 5a). The amount of 5-HT immunoreactivity was lowest at 3 mm cranial to the injury epicenter (9±1) but progressively increased up to 1 mm cranial to the injury epicenter (14±3, Fig. 5b). Immediately caudal to the injury epicenter, the labeling of serotonergic projections decreased again (9±1) followed by a slight increase up to 3 mm caudal to the injury epicenter (12±2, Fig. 5b). In contrast, hMNP-transplanted animals consistently contained a higher degree of 5-HT immunoreactivity in regions corresponding to the location of transplanted cells. hMNP-transplanted animals consistently contained aberrant projections throughout the deeper laminae of the dorsal horn and dense innervation of the ventral horns (Fig. 5a, arrows). At 2 mm and 3 mm cranial to the injury epicenter, and 1 mm caudal to the injury epicenter, 5-HT immunoreactivity was significantly greater than that observed in vehicle-injected animals (17±2 vs. 12±1, p<0.05; 18±1 vs. 9±1, p<0.001; 14±1 vs. 9±1, p<0.05, respectively). At 1 mm cranial to the injury epicenter, and 2 mm and 3 mm caudal to the injury epicenter, 5-HT immunoreactivity was not significantly different than in vehicle-injected animals (p>0.5).

hMNP transplantation enhanced survival of endogenous neurons (Fig. 5c, d). Serial sections were labeled for human nuclei to ensure no human neurons were included in the NeuN cell counts. Cranial to the injury epicenter, vehicle-injected animals had an average of 865±80 endogenous neurons whereas hMNP-transplanted animals had an average of 1091±51 endogenous neurons (p<0.05). Caudal to the injury epicenter, vehicle-injected animals had an average of 871±51 endogenous neurons whereas hMNP-transplanted animals had an average of 1098±57 endogenous neurons (p<0.01).

**Figure 6:**
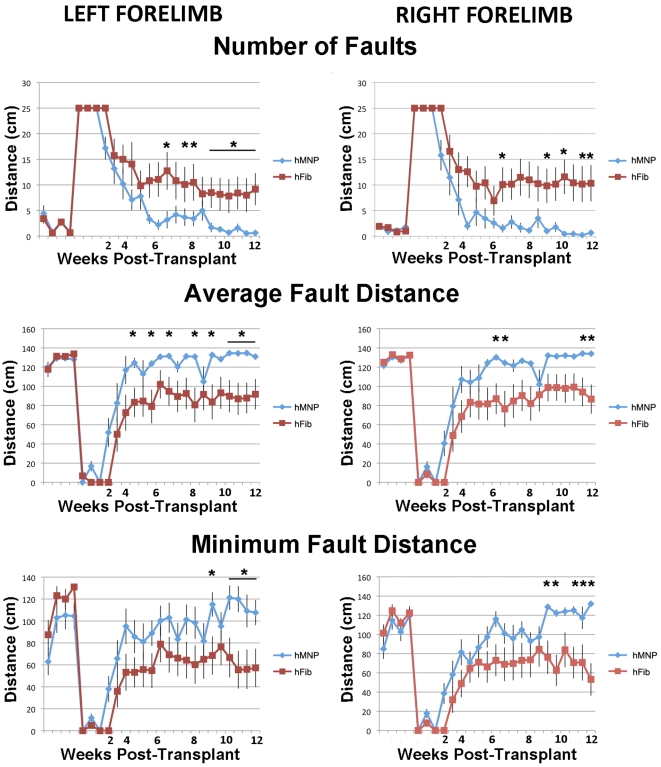
Transplanted hMNPs caused functional benefit. hMNP-transplanted animals performed significantly better than controls on the balance beam, demonstrating fewer foot faults, a greater average fault distance, and an earlier onset of functional recovery as determined by repeated measures ANOVA (p<0.05); * denotes Student's t-test p<0.05.

hMNP transplantation enhanced gross tissue sparing. Morphometric analyses revealed a significantly greater amount of spared tissue in hMNP-transplanted animals compared to vehicle controls, both rostral (7.4±0.5 mm^2^ versus 5.44±0.58 mm^2^) and caudal (8.13±0.6 mm^2^ versus 5.67±0.34 mm^2^) to the injury epicenter. There was no significant difference in tissue sparing between hMNP-transplanted and hFib-transplanted animals. This latter finding indicates that increases in neuronal survival, serotonergic innervation and functional recovery (see below) observed in hMNP-transplanted animals were not due to tissue sparing, as they were absent in hFib-transplanted animals (Fig. 6). This finding is similar to previous reports comparing hESC-derivates and hFib transplant controls [5].

hMNP transplantation altered intracellular signaling pathways (Fig. 5e,f). 1 day following transplantation, we observed an 86.3% decrease in phosphorylation of stress-associated protein kinase (SAPK) in hMNP-transplanted animals (p<0.001). This decrease was maintained at 4 days post-transplant where hMNP-transplanted animals had 59.4% less phosphorylation of SAPK than controls (p<0.05). At 7 days post-transplant SAPK phosphorylation increased in hMNP-transplanted animals, however, no significant difference was observed between groups. Similarly, at 10 days post-transplant the two groups equilibrated and SAPK phosphorylation was not significantly different. As inflammatory molecules (eg. TNFα and CD40) and cellular stressors activate SAPK, it is possible that hMNP-secreted factors inhibited upstream activators of SAPK thus, attenuating downstream effects such as apoptosis, immune activation, and inflammation. PCR array analysis showed that hMNP express 15 fold less CD40 than hFib, in addition to other downregulated pro-apoptotic and inflammatory genes. Therefore, it is possible that transplantation of hMNP alters the immune response following SCI.

### Transplantation of hMNPs Improved Functional Outcome

hMNP transplantation enhanced performance on a balance beam task, which assesses balance and coordination (Fig. 6). hMNP-transplanted animals performed significantly (p<0.01, repeated measures ANOVA) better than hFib-transplanted animals on the balance beam, demonstrating fewer foot faults (0.7±0.3 left limb and 0.6±0.4 right limb in hMNP transplants vs. 10±3.5 left limb and 9.2±3.1 right limb in hFib-transplanted), a greater average fault distance (131±1.7 left limb and 133.9±0.7 right limb in hMNP transplants vs. 91.2±8 left limb and 86.7±15.2 right limb in hFib-transplanted), and a greater minimum fault distance (107.6±11.5 left limb and 131.9±2.2 right limb in hMNP transplants vs. 57.3±17.5 left limb and 53.2±16.8 right limb in hFib-transplanted). hMNP-transplanted animals began to recover one week earlier than hFib-transplanted animals on all parameters of the task. Both groups began to plateau at about 6 weeks post-transplant.

hMNP transplantation enhanced performance on a Montoya Staircase apparatus, which assesses skilled forelimb reaching. hMNP-transplanted animals performed significantly (p<0.01) better than hFib-transplanted animals on number of pellets taken with the right forelimb at 5 weeks (6.5±2 vs. 2±1) and 7 weeks (8±2 vs. 3±1) post-transplant. No differences were observed in left forelimb function. Similarly, hMNP-transplanted animals performed significantly (p<0.01) better than hFib-transplanted animals on the success rate (pellets taken/pellets eaten) of the right forelimb at 5 weeks (15±6% vs. 0%) and 7 weeks (22±9% vs. 2±2%), and of the left forelimb at 7 weeks (27±7% vs. 5±3%) post-transplant. No differences were observed in left forelimb success rate at 5 weeks post-transplant.

hMNP transplantation did not enhanced performance on a grip strength task, which assesses distal limb strength. There was no significant difference (p>0.01) in grip strength of hMNP-transplanted animals compared to hFib-transplanted animals at any time point.

## Discussion

This study directly assessed the potential of a cell-based therapeutic to confer neurotrophic benefit to SCI in the absence of phenotypic benefit. hMNP transplants did not extend processes through myelinated central and peripheral nervous tissue to reinnervate distant muscles, thus, their neurotrophic benefit to the injured spinal cord could be investigated in isolation from the typical phenotypic benefits conferred by a mixed population transplant, such as oligodendrocyte-mediated remyelination or astrocyte-mediated ionic or metabolic homeostasis. These findings further our understanding of the neurotrophic benefits of hMNP transplantation, and are the first to demonstrate anatomical and functional benefit following transplantation of a high purity hMNP population to adult SCI.

Cell-based therapeutics have proven successful in pre-clinical SCI models [14] due to their ability to address multiple features of SCI such as cell loss, demyelination, or homeostatic loss. Several cell replacement strategies have emerged to treat SCI, including O-2A progenitors [15], [16], Schwann cells [17], [18], [19], neural stem cells [8], [20], oligodendrocyte progenitors [4], [5], [21], or bone marrow stromal cells [22]. The ability of bone marrow stromal cells to improve the outcome of SCI exemplifies the importance of graft-derived neurotrophins. In addition, we have recently shown that human oligodendrocyte progenitor cells secrete a number of neurotrophic factors and chemokines that benefit the outcome of SCI, in the absence of graft-derived remyelination [4]. Although the literature on growth factor secretion by motor neurons is limited, it has recently been shown that motor neurons not only respond to neurotrophic cues but also express and secrete growth factors [23]. Our findings confirm a neurotrophic role for hMNPs, verifying that they both expressed and secreted neurotrophic factors that promoted axonal growth and protected neurons from cell death. Specifically, hMNPs expressed NT-3, NT-4, and NGF, which, have all been shown to have beneficial effects following SCI [24], [25]. In addition, hMNPs expressed VEGF, a growth factor that is widely used as an experimental treatment for amyotrophic lateral sclerosis and promotes motor neuron survival [26]. Our data demonstrate that hMNP-CM enhanced axonal elongation and branching of cortical neurites, as evidenced by an increased number of axon collaterals and dendritic arborizations. Trophic effects were also observed in axotomized neuronal cultures, where hMNP-CM enhanced regeneration and sprouting of severed axons. In addition, hMNP-CM significantly enhanced neuronal survival in a neurotoxic environment *in vitro*.

Our transplantation study further confirms that hMNPs have neurotrophic effects on the surrounding environment. hMNP transplantation into SCI sites had beneficial effects on injury pathogenesis, as survival of endogenous neurons and axons was greater in transplanted animals. These effects may be either direct or indirect (non-cell autonomous), as trophic support of glial cells and/or modulation of the glial response may have contributed to neuronal survival and axonal sparing/regeneration. Although many of the identified growth factors have been implicated in neuronal survival, the mechanisms underlying their interaction with the endogenous environment is yet to be elucidated. It has been shown that levels of the p75 neurotrophin receptors (NTR) are upregulated in neurons, while there is a persistent down-regulation of tyrosine-related kinase receptors (TrkRs) in the injury vicinity [27]. Although the role of the p75^NTR^ is highly debated, it predominantly binds the precursor pro-forms of neurotrophins and has been implicated in apoptosis [28]. It is possible that the over-expression of cleaved, mature forms of growth factors by the transplanted cells is enough to prevent, inhibit or counteract neurotrophic action at the p75^NTR^ by enhancing activation and/or upregulation of TrkRs, enhancing downstream neurotrophic effects. Moreover, it is possible that transplantation alters the immune response following SCI, resulting in less immune-related cell death. Although the mode of action is not yet determined, our *in vitro* and *in vivo* data indicate that hMNP-derived growth factors enhance neuronal survival in neurotoxic environments.

In addition to enhancing neuronal survival following SCI, hMNP transplantation enhanced serotonergic innervation around the transplant site. As different ascending and descending axonal projections have been shown to respond preferentially to distinct trophic factors [29], we consider the transplant-induced increase in serotonergic fiber content in the spinal cord a surrogate marker for growth factor-mediated sprouting. As hMNPs secrete a variety of neurotrophins, it is likely that they act on numerous ascending and descending axonal populations.

The changes in SCI pathogenesis following hMNP transplantation correlated with changes in functional recovery. Transplanted animals had an earlier recovery rate of balance and coordination as well as skilled forelimb movements, suggesting an immediate neuroprotective effect, preventing neurons from cell death and axotomized axons from dying back. As transplanted animals recovered to a greater degree, the cells may have acted as a sustained vehicle for neurotrophic release, enhancing sprouting/regeneration of severed descending fibers and possibly restoring connections to endogenous neurons. We did not observe significant differences in forelimb grip strength, as others have reported following Schwann cell transplantation into cervical spinal cord injuries [18]. This discrepancy may be due to the different injury methods, the different cell type transplanted, the different number of cells transplanted, or the different placement of the cells with respect to the lesion epicenter. Nonetheless, the significant improvement in balance and coordination observed in our study is comparable to the functional outcomes observed following transplantation of other cell types into cervical spinal cord injuries [30], [31].

Maturation of transplanted hMNPs was restricted to the ventral horn. The failure of hMNPs to mature in all other regions of the spinal cord likely reflected the gliogenic nature of the SCI environment. Adult spinal cord progenitor cells are restricted to a glial lineage *in situ*, generating predominantly oligodendrocyte precursors [32]. When isolated and expanded *in vitro*, however, adult spinal cord progenitor cells are multipotent and differentiate into neurons, oligodendrocytes, and astrocytes [33]. Nonetheless, upon transplantation into the spinal cord these multipotent progenitor cells largely differentiate into glia [6], [7] while transplantation into neurogenic regions of the brain results in the formation of neurons [6]. Proliferating progenitors following SCI do not express neurogenic basic helix-loop-helix factors or generate new neurons, suggesting that the mechanisms for neuronal differentiation are down regulated [34]. Therefore, it is proposed that the adult spinal cord represents a gliogenic niche. Furthermore, the gliogenic nature of the adult spinal cord is upregulated following injury. Neuron-restricted precursor cells differentiate into mature neuronal subtypes when transplanted into adult non-injured spinal cord, but fail to differentiate when transplanted into injured spinal cord [35]. Likewise, NSCs primed to become cholinergic neurons generate predominantly neurons and region specific cholinergic neurons when grafted into the non-injured spinal cord [36], but generate predominantly glial cells when grafted into the injured spinal cord [37]. Thus, the gliogenic nature of the injured spinal cord poses a significant challenge to neuron replacement strategies.

We conclude that the inherent trophic activity of transplanted hMNPs is sufficient to produce neuronal survival and neurite branching, which correlates with functional recovery observed in transplanted injured animals. Efforts to mature transplanted hMNPs, and enhance axonal projection to target muscles, are important to maximize the benefits of this cell population.

## Materials and Methods

### Ethics Statement

All animal work was carried out in accordance with the UCI Institutional Animal Care and Use Committee (2007–2725). Animals received appropriate post-surgical care including subcutaneous saline, prophylactic Baytril (2.5 mg/kg/d, s.c.; Bayer, Shawnee Mission, KS), and Buprenorphine (0.025 mg/kg/d, s.c.; Western Medical Supply, Los Angeles, CA) for three days. Animals were inspected for weight loss, dehydration, discomfort, and autophagia, with appropriate veterinary care as needed. Research involving human participants was approved by the UCI Institutional Review Board (2008–6467) and the UCI Medical Center with written informed consent. All work involving human embryonic stem cells was approved by the UCI Human Embryonic Stem Cell Research and Oversight Committee (2007–5645).

### hMNP Differentiation

hMNPs were derived from hESC lines hCSC14, hCSC14-CL1 (California Stem Cell, Inc., Irvine, CA) at passages 15–17 and H7 at passages 26–38. hESCs were expanded on Matrigel (BD Biosciences, San Jose, CA) for 1–3 weeks in EctoBias media (California Stem Cell, Inc., Irvine, CA) supplemented with 20 ng bFGF/ml (Millipore, Billerica, MA). Cells were then transferred to ultra low binding dishes (Corning, NY) and suspended in MN differentiation media, which consisted of osmolarity adapted DMEM:F12 mixture (260 mOsm) supplemented with Glutamax, B27 supplement (Gibco-Invitrogen, Carlsbad, CA), insulin 10 µg/ml, sodium selenite 1 ng/ml, transferrin 10 µg/ml (Sigma Aldrich, St. Louis, MO), MgSO4 0.5 mM and bFGF 5 ng/ml (Millipore, Billerica, MA). Cells were exposed to this media for 5 days, supplemented with 10 µM retinoic acid (RA; from 20 mM stock solution in DMSO, Sigma-Aldrich, St. Louis, MO). Cultures were fed daily for the duration of RA treatment, then every 2 days for 16 subsequent days. At day 21, cell clusters were transferred to adherent laminin substrate (20 µg/cm^2^). At day 28, the conditioned media was collected for *in vitro* assays, the cells were prepared for transplantation, or cultured for electrophysiological assessment. Subsets of cells were plated onto matrigel coated 4-well chamber slides (Nunc; Fisher Scientific, Pittsburgh, PA) for immunocytochemical profiling and others were kept for real-time PCR analysis of neuronal markers.

### Electrophysiology

hMNPs were matured for 8 weeks after day 28 on glass coverslips coated with poly-l-lysine and laminin, in the absence of growth factors. Cells were current clamped and a 200 msec, −20 pA conditioning pulse followed by a 1 sec 20 pA step was used for stimulation. The following symmetrical solutions were used for glutamate-mediated stimulation: external solution (mM): KCl 145, CaCl_2_ 2, HEPES 10, D-Glucose 5 pH 7.4 NaOH; pipette solution (mM): KCl 145, CaCl_2_, HEPES 10, EGTA 10 pH 7.2 KOH. Free Ca^2+^ approx 100 nM. Glutamate was applied at 100 µM, as in [38].

### Glutamate-Mediated Toxicity

A stock solution of 100 mM glutamic acid was diluted to either 500 µM or 2000 µM. These dilutions, or 0 µM glutamic acid, were applied to hMNPs in wells for 24 hours before performing cell viability assessment. Propidium iodide (PI) and Annexin V were used to visualize the dead or apoptotic cells on different plates. A stock staining solution of PI and Hoechst was made by adding 15 µl Hoechst and 15 µl propidium iodide to 720 µl PBS. For staining, 10 µl of the PI and Hoechst staining solution was added to each well for 30 minutes at room temperature. Wells were imaged immediately after the incubation using the ImageXpress imager (Molecular Devices). A stock staining solution of Annexin V and Hoechst was made by adding 40 µl Hoechst and 100 µl Annexin V-A647 to 2000 µl PBS. For staining, 20 µl of the stock staining solution was added to each well for 30 minutes at room temperature. Following incubation, cells were fixed with 8% PFA for 15 minutes. The cells were then washed with PBS three times for 5 minutes each. The wells were imaged using the ImageXpress imager (Molecular Devices). PI and Annexin values were obtained from 8 separate wells, averaged, and presented as a percentage of Hoechst positive cells.

### hMNP Growth Factor Secretion

To investigate the growth factor expression profile of hMNPs, PCR was used to detect mRNA and western blots were used to analyze protein expression in both cell lysates and the supernatant. For PCR analysis, hMNPs were trypsinized, spun at 1500×g for 5 minutes, and resuspended in Trizol reagent (Invitrogen, Carlsbad, CA). RNA extraction was carried out according to the manufacturers' protocols. Reverse transcription and PCR were performed using Retroscript RT kit and GeneAMP RNA PCR kit (Ambion, Austin, TX) according to manufacturer recommendations. Samples were run on a 1% agarose gel and water was used as a no-template negative control. In addition, real-time PCR was used to quantify differences in expression between hMNPs and human fibroblasts (hFib, used as a cellular control) using SuperArray neurotrophin and receptors kit (SABiosciences, Frederick, MD). Samples were run in triplicate and data is expressed as fold change versus hFib controls using the delta Ct method. For western blot analysis, conditioned media was collected from hMNPs on day 28 of the differentiation protocol. The media was desalted and concentrated using a YM-10 Centriprep ultrafiltration system (Millipore, Billerica, MA). Proteins were precipitated with a pyrogallol-red molybdate reagent as previously described [39] and immediately resuspended in loading buffer. hMNPs were trypsinized, spun at 1500×g for 5 minutes, and the pellet was resuspended in M-PER (Mammalian-Protein Extraction Reagent; Pierce, Rockland, IL) tissue extraction buffer containing complete-mini protease inhibitor (Pierce, Rockland, IL). Cell lysates (25 ug) and proteins extracted from the supernatant (30 ul of concentrated protein suspension) were loaded onto a 4–12% bis-tris gel (Bio-Rad, Hercules, CA) with a multi-mark molecular weight ladder (Invitrogen, Carlsbad, CA). Proteins were probed with antibodies against NT-3, NT-4, NGF (1∶1000, Millipore, Billerica, MA), and VEGF (1∶2000, AbCam, Cambridge, MA).

### Primary Cortical Cultures

Mixed primary cortical cultures were prepared from postnatal day 1 Sprague-Dawley rats for neurite outgrowth and from embryonic day 18 rat pups for survival and regeneration studies, with and without hMNP-conditioned media (CM) or MN differentiation media (without conditioning). The cortex was mechanically dissociated, trypsinized, and spun at 1500×g for 5 minutes. The pellet was resuspended in Neurobasal media (Invitrogen, Carlsbad, CA). 40,000 cells were plated onto poly-l-lysine coated chamber slides for neurite outgrowth and survival studies and 10,000 cells were plated into the somal side of microfluidic culture platforms coated with poly-l-lysine at 1 mg/ml for neurite regeneration [12]. After seeding, the chamber slides and the microfluidic chambers were fed for the next 2 days with Neurobasal media supplemented with 10 ng/ml FGF until neuronal attachment.

### Neurite Outgrowth Assay

To determine whether hMNP-CM had neurotrophic effects on neurite outgrowth, media was conditioned for 48 hours in the presence of hMNPs and removed for presentation to cortical neurons. Primary cortical neurons were fed with either MN differentiation media or hMNP-CM diluted 1∶1 with MN differentiation media (n = 4/group). For antibody inhibition experiments, neutralizing antibodies against candidate growth factors were added to the culture media at their ND50. Antibodies against neurotrophin 3 (NT 3), neurotrophin 4 (NT4), nerve growth factor (NGF) (Millipore, Billerica, MA), vascular endothelial growth factor (VEGF) (AbCam; Cambridge, MA), and glial derived neurotrophic factor (GDNF) (R&D Systems; Minneapolis, MN) were added to determine whether inhibition of these growth factors would attenuate neurite elongation (n = 4/antibody/group). Primary neurons were fixed on day 7 with 4% paraformaldehyde, immunostained using an antibody against Tuj1 (β-3 tubulin subunit, Covance, Princeton, NJ) and counterstained with Hoechst 33258 (Invitrogen Carlsbad, CA). Twenty pictures at 400× magnification were digitally recorded and used to quantify mean neurite length, the number and types of neurites, and the number of surviving cells. Neurite length was measured by tracing two Tuj1 positive axons per field from the cell body to the tip of the axon using NIH ImageJ software (ImageJ software U. S. National Institutes of Health, Bethesda, Maryland). Neurite length was averaged for each group and expressed in micrometers.

### Neurite Regeneration Assay

To determine whether hMNP-CM had neurotrophic effects on neurite regeneration, media was conditioned for 48 hours in the presence of hMNPs and removed for presentation to axotomized cortical neurons. Primary cortical neurons were plated onto a microfluidic culture platform that isolates axons from somata [12]. After 7 days of axon growth and maturation, the media and axons were aspirated using a fire-polished glass pipette. Axotomy was confirmed under a microscope and the media was replaced with either hMNP-CM or MN differentiation media (n = 5/group). Axons were allowed to regenerate for 5 days, the cultures were fixed in 4% paraformaldehyde and stained for neurofilament (Millipore, Billerica, MA) and MAP-2 (Sigma-Aldrich, St. Louis, MO). 400× images were taken along the length of the neurite side of the platform using a confocal microscope, inverted to grey scale images of the same threshold, and optical density was determined as mean grey value in pixels using Image J software (U. S. National Institutes of Health, Bethesda, Maryland, USA) and averaged across groups.

### Cytotoxicity Assay

Following CNS injury, activated microglia release toxic substances such as nitric oxide, tumor necrosis factor alpha, and interleukin-1β that play an essential role in neuroinflammation and neurodegeneration [40], [41]. To investigate whether hMNP-CM can protect cortical neurons from the neurotoxic effects of activated microglia, BV-2 microglia were cultured and the conditioned media was collected after 24 hours from control microglia and microglia activated with 100 ng/ml of lipopolysaccharide (LPS; kind gift from Dr. Frank LaFerla's Lab). Microglial conditioned media (MG-CM or LPS-MG CM) was then filtered and used for neuronal survival assays in the presence of hMNP-CM or MN differentiation media (n = 4/group). After neuronal attachment, the cultures were treated with either MG-CM or LPS-MG CM diluted 1∶1 with hMNP-CM or MN differentiation media. After 3 days of treatment, cultures were fixed in 4% paraformaldehyde, immunostained for MAP-2 (Sigma-Aldrich, St. Louis, MO) and 20 digital images of each chamber well were captured at 400× magnification. The number of positive neurons was quantified using ImageJ software and the mean number of neurons for each group was recalculated as cells/cm^2^.

### Myoblast and hMNP Co-Culture

Muscle fragments from day 1–5 rats or adult human biopsies were minced and dissociated using 2 mg/ml Collagenase IV (Invitrogen, Carlsbad, CA) and transferred in a 25 cm^2^ cell culture flask coated with Matrigel 1∶30 (BD Biosciences, San Jose, CA). Cultures were fed every second day with MyoBlast media (California Stem Cell Inc., Irvine, CA) supplemented with 10 ng/ml FGF2 (Millipore, Billerica, MA).

hMNPs and myoblasts were transferred to separate chambers of microfluidic culture platforms connected by 5–20 µm micro-channels [12]. The neural chamber was seeded with hMNPs and cultured for 2 weeks in serum-free MN differentiation media supplemented with 2 ng/ml FGF2. The muscle chamber was then seeded with myoblasts and fed with MyoBlast media (California Stem Cell Inc., Irvine, CA) for two days followed by MyoBlast media supplemented with 10% FBS. After 2–3 days, a video camera attached to the microscope was used to record the muscular activity.

### Immunocytochemical Labeling

Cultures were fixed, blocked, and exposed to the primary antibodies anti-PAX6 1∶200, anti-NG2 1∶200, anti-serotonin 1∶200, anti-Olig1/2 1∶200, anti -Isl1 1∶200, anti-PDGFR-2 1∶100, anti-Tuj1 1∶1000, anti-human nuclear antigen 1∶200, anti-synaptophysin 1∶100, anti-SMI-32 1∶1000, anti-ChAT 1∶100 (Millipore, Billerica, MA) and anti-HB9 1∶100 (MNR2 Ab from DSHB Developmental Studies Hybridoma Bank) at 4°C overnight. Primary antibody application was followed by fluorescent secondary antibodies (CY3 or Alexafluor-488 conjugated; Invitrogen, Carlsbad, CA).

To immunocytochemically assess MN functionality; Co^2+^ labeling was performed [42], [43]. Cultures were exposed to 100 µM kainite plus 2.5 mM CoCl_2_ in uptake buffer (139 mM sucrose, 57.5 mM NaCl, 5 mM KCl, 2 mM MgCl_2_, 2 mM CaCl_2_, 12 mM glucose, 10 mM Hepes) for 10 minutes at room temperature, washed in uptake buffer plus 2 mM EDTA and incubated in 0.05% (NH_4_)_2_S for 5 minutes. After two washes in PBS, cells were fixed in 4% paraformaldehyde for 1 hour. A modified Timm's stain procedure was employed at room temperature [44], [45]. The cultures were incubated in the dark in a solution consisting of 1 part solution A (0.1 M AgNO_3_), 20 parts of solution B (2% hydroquinone and 5% citric acid in water), and 100 parts of solution C (20% gum arabic, in water).

For neurite and neuronal cell body identification, fixed cortical neuron cultures were blocked, and then exposed to primary antibodies (anti-neurofilament 70; 1∶500 (Millipore, Billerica, MA), anti-MAP-2; 1∶1000 (Sigma, St. Louis, MO) at 4°C overnight, followed by fluorescent secondary antibodies (Alexafluor – 488 or 594 conjugated; Invitrogen, Carlsbad, CA).

For neuromuscular junction identification, myoblast and MN co-cultures were fixed with 4% paraformaldehyde for 10 minutes, and then exposed to α-bungarotoxin, Alexa Fluor 594 conjugate (Invitrogen, Carlsbad, CA) for 1 hour.

### Spinal Cord Injury Model

Adult, female Sprague-Dawley rats (Charles River Laboratories, San Diego, CA) were anesthetized using Ketamine (100 mg/kg) and Xylazine (10 mg/kg, i.p.: Western Medical Supply, Los Angeles, CA) and underwent a C5–C6 laminectomy. A 200 kD bilateral contusion injury was delivered using an Infinite Horizons Impactor (Precision Systems and Instruments, Lexington, KY). Seven days later, the animals were re-anesthetized and the laminectomy site was re-exposed. The animals were secured to a stereotactic apparatus, and a total of 100,000 hMNPs, 500,000 hMNPs, human fibroblasts (hFib) (n = 15/group, 25,000 cell/µl) or volume equivalent of vehicle (n = 15) were transplanted bilaterally into the ventral horn (0.5 mm from the midline, 1.2 mm from the dorsal surface) rostral and caudal to the injury site using a 33 G needle attached to a Hamilton syringe. One day prior to transplant and for the duration of the experiment, animals received cyclosporine A (20 mg/kg/d, s.c.; Bedford Laboratories, Bedford, OH).

Animals were acclimated 10 minutes everyday for one week, then trained on the balance beam (Lafayette Instruments, Lafayette, IN) for 2 weeks prior to injury. Following injury, the animals were tested two times a week. For testing, animals traversed a 135 cm, tapered balance beam 5.5 cm wide at the start and 1.5 cm wide at the finish. The beam is equipped with a 2 cm wide ledge that is attached 2 cm below the surface of the beam to provide support for injured animals [46]. The animals ran 5 trials with a 1minute break in between each trial and the number of foot faults and foot fault distance was recorded for the left and right forelimb by two independent observers. Animals that could not perform the task following SCI received the maximum number of foot faults/trial (5) and a zero as their fault distance.

Grip strength assessments were performed on animals trained to the task everyday for 3 weeks prior to SCI. Baseline data was collected on the last four days of training before SCI. Animals were tested once a week for 6 weeks and every other week thereafter. The testing period consisted of 5 trials where the animals were presented to the grip strength meter bar (TSE-Systems and distributed by SciPro, Inc.) while one forelimb was restrained by the experimenter. The force with which the animals' grabbed the bar was recorded for each trial and for each forelimb, averaged, and averaged across groups.

Skilled reaching was assessed using the Montoya Staircase apparatus modified to increase the sensitivity using color-coded pellets [47]. The animals were trained for 3 weeks where they were placed in the plexiglass staircase for fifteen minutes and allowed to grab freely at the different color pellets baited on each stair. Baseline data was collected 3 days prior to injury and post-injury data collection was performed twice a week for fifteen minute testing sessions and averaged. The number of pellets taken and the number of pellets eaten were recorded and used to determine the success rate.

### *Smn^−/−^;SMNΔ7^+/+^;SMN2^+/+^* Model

*Smn^−/−^;SMNΔ7^+/+^;SMN2^+/+^* mice at post-natal day 1 were secured to a stereotactic apparatus and a total of 20,000 hMNPs (n = 15/group, 10,000 cell/µl) or volume equivalent of vehicle (n = 15) were transplanted bilaterally within the 6^th^ thoracic segment using a 33 G needle attached to a Hamilton syringe.

### Immunohistochemical Labeling

For the SCI model, a small cohort of spinal cord injured animals in each group (n = 4) received intramuscular injections of the retrograde tracer, cholera toxin B (1 mg/ml CTB; Invitrogen, Carlsbad, CA), to label innervating motor axons four days prior to sacrifice. The animals received 4-1 ul injections into the bicep muscle and 8-1 ul injections into the triceps muscles using a 10 ul Hamilton syringe. 2 or 3 months post-transplant, the animals were sacrificed by aortic perfusion with 4% paraformaldehyde and the tissue was cryopreserved for sectioning. Tissue was immunolabeled for neuronal nuclei (NeuN; 1∶200, Millipore, Billerica, MA), oligodendroglial cells (OSP; 1∶1000, AbCam, Cambridge, MA), astrocytes (GFAP; 1∶1000, DAKO, Carpenteria, CA), interneurons (GAD65/67; 1∶1000, AbCam, Cambridge, MA), neuronal processes (Tuj1; 1∶1000, Covance, Princeton, NJ), retrograde transport (CTB, Invitrogen, Carlsbad, CA) or the motor neuron lineage markers Islet1 (Isl-1; 1∶200, AbCam, Cambridge, MA) and ChAT (1∶100; Millipore, Billerica, CA) using immunofluorescence or the ABC method with DAB+nickel as a substrate.

NeuN positive neurons were quantified in sections 200 µm apart at 2 mm rostral and 2 mm caudal to the injury epicenter. Serial sections were labeled with human nuclear antigen to ensure no human cells were included in the analysis. Images were captured at 400× magnification from 4 fixed regions within the grey matter representing >30% of the total grey matter (n = 5 animals/group). The number of cells in each area was quantified using ImageJ software, averaged for each animal, and recalculated as neurons per mm^2^. To investigate whether transplantation of hMNPs enhanced axonal sprouting of descending and ascending tracts, we visualized descending serotonergic projections (5-HT, 1∶200; AbCam, Cambridge, MA). Digital images were captured of both dorsal and ventral horns at 200× magnification (for 5-HT) and dorsal horns only (for CGRP) from tissue sections spaced 200 um apart (n = 5 animals/group). The area quantified represented >50% of the total gray matter area. All pictures were taken at the same exposure, imported using ImageJ software (U. S. National Institutes of Health, Bethesda, Maryland) and inverted to grayscale images of the same threshold. The density of staining was determined by multiplying the grey value in pixels by the area measured and was expressed in arbitrary units (au). Statistical analysis was performed using a Student's t-test. Morphometric analysis of spinal cord size was conducted by tracing three tissue sections 100 µm apart 1 mm cranial and 1 mm caudal to the injury epicenter, using Olympus MicroSuite B3SV software; the measurements within corresponding blocks from animals within a group were then averaged. All cystic enlargements were subtracted from the total area.

For the *Smn^−/−^;SMNΔ7^+/+^;SMN2^+/+^* model, mice were sacrificed 7 days post-transplantation via trans-cardiac perfusion with 4% paraformaldehyde (*Smn^−/−^; SMNΔ7^+/+^;SMN2^+/+^* mice typically expire within 14 days of birth). The tissue was cryopreserved in 27% sucrose and the cords were sectioned into 1 mm blocks and embedded in OCT compound. Tissue was cryosectioned at 20 µm and mounted onto gelatin coated slides. Tissue sections were blocked with 10% NDS in PBS and permeabilized with 0.1% Triton X-100. To determine the fate of the transplanted hMNPs, sections were double-labeled with primary antibodies Ku80 (1∶300; Abcam) and Islet-1 (1∶200; Abcam) overnight at room temperature. Donkey anti-rabbit conjugated with biotin (1∶250; Invitrogen), Streptavidin 594 (1∶250; Invitrogen), and Streptavidin 488 (1∶250; Invitrogen) or goat anti-rabbit Alexa Fluor 488 (1∶250; Invitrogen) was used for 2 hours at room temperature as secondary antibodies.

### Transplant-Induced Spinal Cord Changes

To investigate the signaling pathways that were altered following hMNP transplantation, 200 kD bilateral contusion injuries were induced as above and 100,000 hMNPs (n = 16) or vehicle (n = 16) were injected intraspinally seven days post-injury. All animals were injected with 20 mg/kg/day of CSA. 4 animals from each group were sacrificed at 1, 4, 7, and 10 days post-transplant by decapitation (n = 4/group/timepoint). The spinal cords were quickly removed, flash frozen in liquid nitrogen, and stored on ice in 95% ethanol. Protein was extracted using RIPA buffer (Sigma-Aldrich, St. Louis, MO) supplemented with protein and phosphatase inhibitors at 2× concentration (Pierce, Rockland, IL). The tissue was homogenized and the extract was spun at 13000 rpm for 15 minutes. The supernatant was collected and protein concentrations were determined using BCA assay (Pierce, Rockland, IL). Proteins were loaded onto a 4–12% Bis-Tris gel (Bio-Rad, Hercules, CA) at a concentration of 10 ug. Kaleidoscope molecular weight markers were used as a standard (Bio-Rad, Hercules, CA). Nitrocellulose blots were probed for MAP kinases (Cell Signaling, Danvers, MA) at a dilution of 1∶1000 according to manufacturer's protocol. Secondary antibodies conjugated to HRP (Cell Signaling, Danvers, MA) were used at a concentration of 1∶2000 or 1∶5000 and Super Signal West Pico chemiluminescent substrate (Pierce, Rockland, IL) was used for detection. Densitometry was performed using ImageJ software (U. S. National Institutes of Health, Bethesda, Maryland) and data is expressed relative to actin control. Significance was determined using a Student's t-test.

## Supporting Information

Figure S1 (a) Real-time PCR revealed a 10-fold increase in NT-3, a 23-fold increase in NT-4, an 18-fold increase in VEGF, a 3.5-fold increase in CNTF, a 6-fold increase in IL10, a 121-fold increase in NRG4 (neuregulin 4), and a 275-fold increase in NPY (neuropeptide Y) when compared to controls. There was a 5-fold decrease in NGF expression in hMNP when compared to hFibs (b) Western blot analysis confirming that hMNP express and secerete NT-3, NT-4, NGF, and VEGF.
(7.37 MB TIF)
