# Supplementary figures and images for: Histological and Functional Benefit Following Transplantation of Motor Neuron Progenitors to the Injured Rat Spinal Cord
Source: PLoS One. 2010 Jul 29;5(7):e11852. doi: 10.1371/journal.pone.0011852 (PMC2912300; doi:10.1371/journal.pone.0011852)

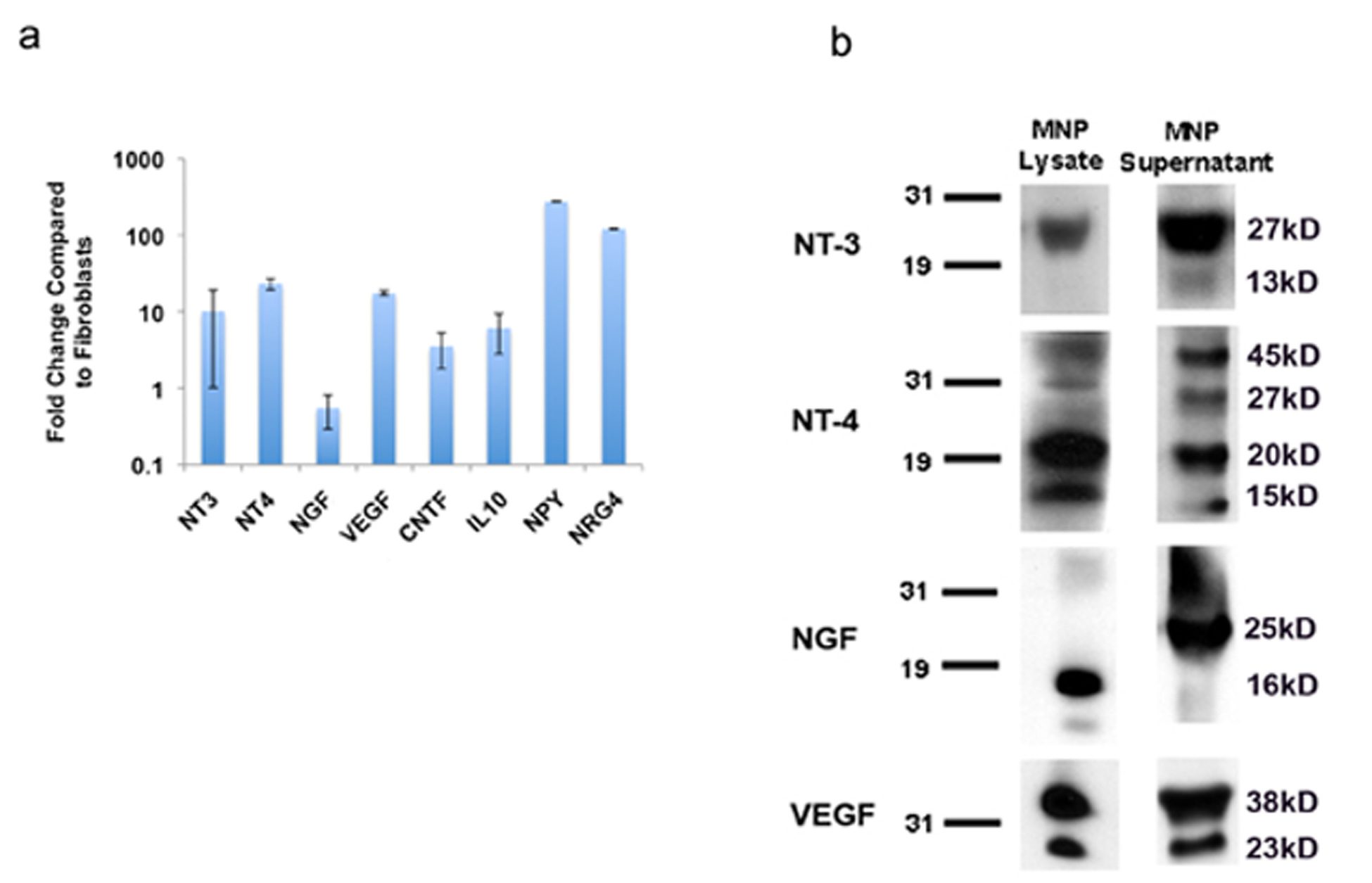

Supplement: Figure S1 — (a) Real-time PCR revealed a 10-fold increase in NT-3, a 23-fold increase in NT-4, an 18-fold increase in VEGF, a 3.5-fold increase in CNTF, a 6-fold increase in IL10, a 121-fold increase in NRG4 (neuregulin 4), and a 275-fold increase in NPY (neuropeptide Y) when compared to controls. There was a 5-fold decrease in NGF expression in hMNP when compared to hFibs (b) Western blot analysis confirming that hMNP express and secerete NT-3, NT-4, NGF, and VEGF. (7.37 MB TIF) [file pone.0011852.s001.tif]
